# Exploring novel Apalutamide analogues as potential therapeutics for prostate cancer: design, molecular docking investigations and molecular dynamics simulation

**DOI:** 10.3389/fchem.2024.1418975

**Published:** 2024-08-06

**Authors:** Ajay Kumar Gupta, Yogesh Vaishnav, Sanmati Kumar Jain, Sivakumar Annadurai, Neeraj Kumar

**Affiliations:** ^1^ Drug Discovery and Research Laboratory, Department of Pharmacy, Guru Ghasidas Vishwavidyalaya (A Central University), Bilaspur, Chhattisgarh, India; ^2^ Department of Pharmacognosy, College of Pharmacy, King Khalid University, Abha, Saudi Arabia; ^3^ Department of Pharmaceutical Chemistry, Bhupal Nobles’ College of Pharmacy, Udaipur, Rajasthan, India

**Keywords:** Apalutamide, prostate cancer, bioisosteric approach, molecular dynamics simulation, molecular docking 1

## Abstract

**Introduction:** Prostate cancer (PC) ranks as the second most frequent type of cancer in men and is the fourth largest cause of mortality worldwide. Androgenic hormones such as testosterone and dihydrotestosterone are crucial for the development and progression of the prostate gland. Androgenic hormones bind to androgen receptors (AR) and trigger the synthesis of many genes that stimulate the growth of prostate cells, initiating PC growth. Apalutamide (APL) is a non-steroidal antiandrogen drug used to treat PC; however, it also causes a variety of toxicities and resistance during the treatment.

**Methods:** The purpose of this study was to computationally identify new and safer analogues of APL, focusing on improved pharmacokinetic properties and reduced toxicity. Drug likeness (DL) and drug score (DS) were also calculated. Docking studies on the designed analogues were conducted to predict their binding affinities and compare their orientations with the ligands in the original crystal structure. Molecular dynamics (MD) simulation of docked ligands was done using Schrödinger suite.

**Results:** We generated a total of 1,415 analogues for different groups of APL using the bioisosteric approach. We selected 80 bioisosteres based on pharmacokinetic profiles, DL and DS score predictions, and found that the designed APL bioisosteres were optimal to good compared to APL. Analogues APL19, APL35, APL43, APL76, and APL80, formed hydrogen bonds with protein (*PDB ID: 5T8E*) which is similar hydrogen bonding to the standard (APL). The MD simulation result confirmed that APL43 and APL80 complexes were stable during the 100 nS run.

**Discussion:** The results suggest that the APL analogues, particularly APL43 and APL80, are predicted to be potential antiandrogen drugs for the treatment of prostate cancer.

## 1 Introduction

The prevention of cancer is a significant priority in public health in the 21st century, considering the increasing worldwide effect of the illness. In 2022, the International Agency for Research on Cancer (IARC) estimated that there would be around 20 million cases of cancer diagnosed and 9.7 million deaths linked to cancer globally. Prostate cancer is second in terms of frequency among all malignancies in men, and fourth overall. In the year 2022, there were about 1.46 million numbers of prostate cancer and 0.39 million deaths (Bray et al., 2021; Chen et al., 2023; Guida et al., 2022; Ferlay et al., 2024; Sung et al., 2021; Ferlay et al., 2021). In India, the incidence of prostate cancer (PC) is projected to increase from 43,691 cases in 2022 to 47,068 cases in 2025. Furthermore, it has been recognized as the second most common form of cancer among males who are 65 years of age or older, with a total of 33,695 cases and a prevalence rate of 12.3% (Sathishkumar et al., 2022; Kulothungan et al., 2022). Androgens, which are male sex hormones, are a group of hormones that regulate the growth and sustain the traits associated with male characteristics. Testosterone and dihydrotestosterone are the most prevalent androgens in males which are necessary for the proper development and operation of the prostate gland. Androgens stimulate the proliferation of both healthy and malignant prostate cells by attaching to and activating the androgen receptor (AR). Upon activation, the AR induces the production of several genes that promote the proliferation of prostate cells (Mohler et al., 2011; Zhang et al., 2016; Stabile and Dicks, 2003). During the early stage of PC, it is often classified into four stages: The early stage, or localized (Stages I and II: When the tumor remains confined to the prostate and has not spread beyond it), locally advanced (Stage III: Cancer has metastasized beyond the prostate but is limited to adjacent tissues) and advanced (Stage IV: Cancer has metastasized beyond the prostate to distant sites such as the lymph nodes, bones, liver, or lungs). A blood test often diagnoses PC by measuring levels of prostate-specific antigen (PSA), with a threshold of PSA >4 ng/mL. In addition, the diagnostic process may also include a digital rectal examination (Belkahla et al., 2022). Metastatic disease, discovered either during the diagnostic process or after local therapy-induced recurrence, causes most PC deaths. Castration-resistant prostate cancer (CRPC) is the last stage of advanced PC that results from the tumor’s adaptability to a low-testosterone environment. It typically takes three to 8 years for individuals to respond to androgen deprivation therapy (ADT), at which point they often start to exhibit symptoms of metastatic CRPC (Harris et al., 2009; Tran et al., 2009; Murray et al., 2022; Huang et al., 2023a).

ADT used to be thought of as the standard treatment for metastatic hormone-sensitive prostate cancer (mHSPC), but in 2015, new information showed that ADT was not always effective and that some patients were actually resistant to it (López-Abad et al., 2024; Gan et al., 2018; Huang et al., 2023b). Antiandrogen drugs are used to treat PC. Flutamide and bicalutamide, which were among the first non-steroidal antiandrogen drugs, showed efficacy in treating PC in their early stages. However, their effectiveness diminished when the disease progressed to a hormone-resistant stage. Flutamide and bicalutamide, in a cases of cancer that do not respond to therapy, function as agonists to stimulate the excessive production of androgen receptors (AR), thereby facilitating the advancement of the disease (Kelly et al., 1997). Consequently, the development of second-generation drugs has prioritized the adjustment of agonist activity while maintaining antiandrogen action in cells that have an excessive amount of AR (Tran et al., 2009; Gao et al., 2021). Apalutamide (APL) is a second-generation nonsteroidal antiandrogen drug or androgen signaling inhibitor (ASI) that was developed by the group of Sawyers and Jung at the University of California, Los Angeles (UCLA). The approval was granted by the United States in February 2018 and by the European Union in January 2019 (Hughes, 2020; Chi et al., 2019). Animal studies indicate that APL is somewhat more effective than enzalutamide and may have a reduced risk of seizures due to its lesser ability to enter the brain, which is four times lesser than enzalutamide (Clegg et al., 2012). By acting as an AR inhibitor, APA stops the nuclear translocation of AR. AR hinders transcription and DNA binding and is hindered by APL. APL demonstrated a significant clinical response in a Phase-II trial treating males with non-metastatic castration-resistant prostate cancer (nmCRPC), with 89% of patients with high-risk nmCRPC seeing a reduction in PSA of at least 50% by the 12-weeks mark (Borno et al., 2019; Shen et al., 2023; Zhu et al., 2023).

APL is quickly absorbed after being taken orally, and it reaches its maximum concentration (T-max) within 2 h. It is metabolised by the enzymes CYP2C8 and CYP3A4 (cytochrome P450) to produce its active metabolite, N-desmethyl apalutamide. After a period of 70 days after the administration of the radiolabeled dosage, it was discovered that 65% of the medicine was excreted in urine and 24% was retrieved in feces (Pérez-Ruixo et al., 2020; Huang et al., 2023c). During the Spartan study, APL causes hypertension to varying degrees. Cardiac toxicities resulted in treatment cessation and, specifically, hypertension and atrial fibrillation in a limited number of patients. During the Titan study, APL also causes hypertension of varying severity. Ischemic heart disease, characterized by a minor abnormality in the QT interval, has also been seen in patients and resulted in the death of two individuals. In the Spartan study, a few individuals had treatment stoppages due to renal toxicities such as hematuria and acute kidney damage. Urinary retention was also seen in participants during the Titan experiment (Belderbos et al., 2018; Lv et al., 2024). Although APL is generally regarded as safe, concerns arise regarding its potential impact on the central nervous system (CNS). CNS effects might be diminished the overall quality of life, result in permanent effects, or required the discontinuation of therapy. However, it is crucial to acknowledge, the spreading of tumors may have significantly contribute to the emergence of neurological symptoms. These characteristics have prompted the publication of several studies about the safety of novel hormonal therapy for metastatic castration-resistant prostate cancer (mCRPC). However, there is little data regarding to the occurrence of seizures and neuropsychiatric symptoms in patients with nmCRPC (Sutil et al., 2021; Slovin et al., 2018; Gillessen et al., 2018; Pilon et al., 2017).

**FIGURE 1:**
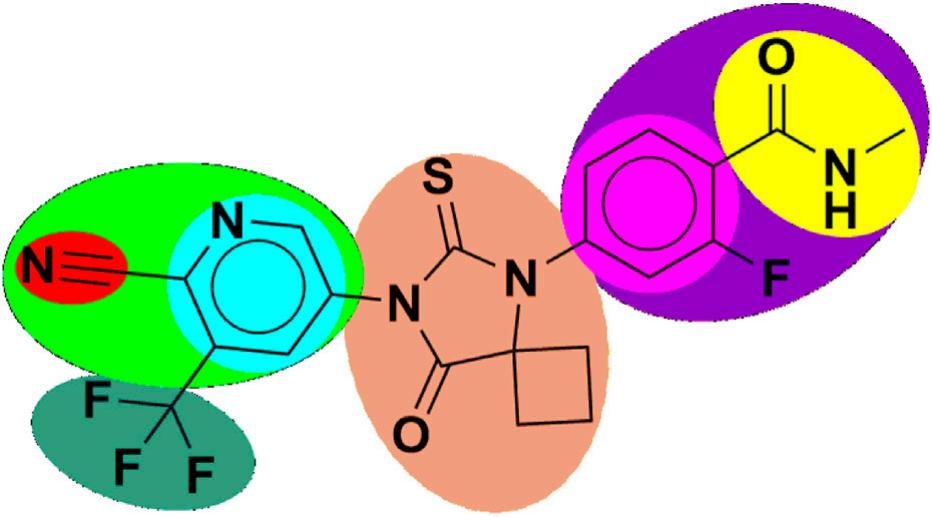
Structure of apalutamide and its modified groups, including cyano (red colour), cyano-pyridinyl (green colour), pyridinyl (cyan colour), cyclobutyl-thiohydantoin (coral colour), phenyl (pink colour), 2-fluoro-N-methylbenzamide (purple colour), N-methyl formamide (yellow colour), and trifluoromethyl (aquamarine colour).

Therefore, it is imperative to change the structure of APL (Figure 1) in order to design newer APL analogues with safer and less toxic antiandrogen drugs for the treatment of PC. In this work, we designed the newer analogues of APL by the use of scaffold-transforming techniques. It is a unique technique in medicinal chemistry for the lucid design of drugs by gradually altering the parent component to develop a variety of different compounds with improved therapeutic potential (Manjunatha et al., 2019; Zheng et al., 2022). The scaffold-transforming techniques (bioisosteric approach) were applied to design APL analogues that exhibit a comparatively higher level of safety, followed by *in silico* pharmacokinetic (ADMET), docking studies and molecular dynamics (MD) simulation.

## 2 Materials and methods

The smile notation of the APL was taken from DrugBank, a widely-utilized chemical information library. In addition, the MolOpt was used to generate the several bioisosteres for the APL molecule. The chemical structures of APL and these analogues were drawn using ChemDraw software. The ADMETLab 2.0 online tool was used to predict the pharmacokinetic and toxicological properties. The Osiris property explorer (PEO) was used to determine the drug likeness (DL) and drug score (DS). The docking investigation was performed using AutoDock Vina (ADV), and the docking results were analyzed using Discovery Studio.

### 2.1 Designing of apalutamide bioisosteres

APL is used as a second-generation antiandrogen medication for the treatment of PC. On the other hand, people receiving this medication often experienced a variety of toxicities. Therefore, it is essential to alter the APL structure to mitigate its hazardous effects. MolOpt is an online program that uses deep generative models, data mining, and similarity comparisons to generate bioisosteres. It utilizes bioisosteric transformation rules. MolOpt has capability to explore the historical bioisosteric group space and discover novel bioisosteric transformation concepts (Shan and Ji, 2020). A total of 1,415 bioisosteres were generated by replacing various groups in the APL molecule. We then subjected these bioisosteres to further screening, including ADMET, DL, DS prediction, and docking investigations.

### 2.2 Pharmacokinetic and toxicological (ADMET) properties predictions

The prediction of ADMET properties of generated APL bioisosteres were computed using ADMETlab 2.0. It is an integrated online platform with eighty-four quantitative and four qualitative regression models with authentic and extensive predictions of ADMET properties for novel ligands that mimic mammalian ADMET properties (Xiong et al., 2021; Dong et al., 2018; Wang et al., 2016; Lei et al., 2016).

### 2.3 Drug likeness (DL) and drug score (DS) prediction

DL and DS evaluations are essential in the first phase of the drug development process. They assist researchers in selecting and prioritizing compounds, allowing them to concentrate on candidates that have a greater chance of success in subsequent development and clinical testing. PEO (Sander et al., 2009) helped to calculate DL and DS.

### 2.4 Molecular docking analysis

Molecular docking is commonly used technique in the field of drug development that aims to find potential lead molecules, enhance their binding interactions, and forecast their binding affinities to particular biological targets. This tool is beneficial for understanding the basic foundation of protein-ligand interactions and designing novel therapeutic drugs (Feher and Williams, 2012; Forli et al., 2016). We performed a molecular docking study to investigate the interactions between the crystal structure of the AR and the designed APL bioisosteres. This work involved several stages, including ligand preparation, protein preparation, and investigation of protein-ligand interactions (Trott and Olson, 2010).

#### 2.4.1 Protein preparation

The three-dimensional structure of protein was retrieved from the Protein Data Bank (PDB) database (https://www.rcsb.org/). The selective androgen receptor modulator (*PDB ID: 5T8E*, Resolution = 2.71 Å) was used as a protein to identify the interaction between ligand and protein binding domain (Asano et al., 2017). The protein was first prepared for docking studies by removing water molecules, adding hydrogen atoms, and adding Kollman charges, followed by the repair of missing atoms, and saved in PDBQT format.

#### 2.4.2 Ligand preparation

ChemDraw was used to draw the 2D chemical structure of the ligands. Chem3D for APL and their designed analogues (ligands) converted the 2D structure into a 3D structure. The ligands were subjected to energy minimization using Chem3D and stored in SDF format. The OpenBabel (O’Boyle et al., 2011) software was used for the conversion into MOL2 format. Then, ligands were introduced into ADV and then stored in PDBQT format for the docking procedure. Furthermore, the introduction of protein into a solvent called ADV facilitates the arrangement of grid boxes, ensuring that the ligand remains in the core.

#### 2.4.3 Molecular docking

We performed the docking simulations between the ligands and the protein using the ADV software. The whole protein’s active site was then the focus of a grid box that had a grid spacing of 1.0 Å and dimensions of size x = 40, size y = 40, and size z = 40. The grid centre was located at X = 23, Y = 7, and Z = 7. The default settings for other docking parameters, including ADV and the rates of crossover and gene mutation, remained in place. The resulting files were analyzed using Discovery studio (Kemmish et al., 2017) to generate 2D and 3D protein-ligand interactions.

### 2.5 Molecular dynamics simulation

The top two complexes, based on docking scores and interactions, were selected for the molecular dynamics (MD) simulation. The MD simulation was conducted on an Acer workstation running Ubuntu 22.04. The Desmond software in the Schrödinger suite is used to run the MD simulation to elucidate the effectiveness of the screened compounds by molecular docking (Alturki et al., 2022; Van Der Spoel et al., 2005). The protein-ligand complexes were prepared using the ‘System Builder'. After reducing its volume, the SPC water model with an orthorhombic shape was selected. It has 10 × 10 × 10 Å periodic boundary conditions in the protein-ligand complex’s x, y, and *z*-axes. Moreover, the androgen receptor modulator protein (*PDB ID: 5T8E*) received additions of 25 sodium ions and 30 chloride ions. Ion and salt placements within 20 Å were excluded from neutralizing the simulation. Also, the complex’s energies were lowered using the OPLS2005 forcefield by heating and reaching an equilibrium state before the MD simulations were run (Banks et al., 2005). We used the steepest descent method-based minimization protocol against the complexes and then heated them at 0–300 K. Further, with the time step of 100 nS, the system normalized into an equilibrium state at 1,000 steps. We kept the final production run for 100 nS at time steps of 100 ps, 300 K temperature, and 1.0325 bar pressure for both complexes, applying the Nose-Hoover method with the NPT ensemble (Huang et al., 2011).

## 3 Results and discussion

### 3.1 Bioisosteres of Apalutamide

Scientists or chemists often use the bioisosteric strategy to enhance pharmacokinetic characteristics and reduce undesired toxicities. MolOpt generated 1,415 replaceable groups for various groups in the APL molecule. The screened compounds are shown in Supplementary Table S1.

### 3.2 Prediction of molecular properties

The prediction of molecular properties for APL bioisosteres were computed and is shown in Supplementary Table S1. The Lipinski rule of five comprises the molecular weight (MW), number of hydrogen bond acceptors (nHA), number of hydrogen bond donors (nHD), and logarithm of partition coefficient value (logP). All analogues meet the Lipinski’s rule of five, indicating appropriate absorption and bioavailability of the drug candidates. So, all analogues may be considered as drug candidates. All analogues exhibited excellent topological polar surface areas (TPSA), suggesting their potential to penetrate cells.

### 3.3 Prediction of medicinal properties

A quantitative estimate of druglikeness (QED) is a property that measures the druglikeness properties of drug candidates. It is based on the idea of desirability, which encompasses eight drug-like-related properties. The QED score of the designed analogues, such as APL32, APL74-76, APL38, APL43, APL75, APL76, APL78-80, and APL18-22, is in the good range (>0.67), but the QED score for APL is 0.538. The QED score shows that all analogues are attractive compounds. MCE-18 is an abbreviation for the concept of medicinal chemistry evolution in the year 2018. This metric is capable of accurately assessing the novelty of compounds based on their overall sp3 complexity. The MCE-18 score of newly designed analogues such as APL1-8, APL9-14, APL15-26, APL27-30, APL32-34, APL45-76, APL79, and APL80 found more than 78, as these analogues need to be visually examined to evaluate their target profile and drug-likeness. Lipinski’s rule [Karami et al., 2022] has been accepted for all analogues, suggesting the potential for proper absorption or permeability. Pfizer’s rule of all analogues were also found under the accepted criteria, with some exceptions, including APL6, APL43, APL69, APL72, APL73, APL79, and APL80, which indicate favorable ADMET profiles. The Golden Triangle (GT) rule comprises of two parameters, including MW (≤200 and ≥50) and LogP (≤5 and ≥ −2). All analogues, except for APL26, APL27-44, APL70, APL71, and APL74, met the acceptance criteria of the GT rule. The calculated medicinal properties of all APL analogues are being in Table 1.

**TABLE 1:** Medicinal Properties, DL and DS of analogues.

Entry no.	QED	Synth	MCE-18	Lipinski	Pfizer	GT	DL	DS
APL1	0.544	3.527	96	Accepted	Accepted	Accepted	−12.82	0.15
APL2	0.512	4.115	124	Accepted	Accepted	Accepted	−11.47	0.17
APL3	0.512	4.115	124	Accepted	Accepted	Accepted	−11.47	0.17
APL4	0.526	3.81	109	Accepted	Accepted	Accepted	−7.65	0.14
APL5	0.529	3.667	96	Accepted	Accepted	Accepted	−10.38	0.14
APL6	0.512	3.571	91	Accepted	Rejected	Accepted	−9.51	0.14
APL7	0.514	3.563	96	Accepted	Accepted	Accepted	−10.48	0.14
APL8	0.439	3.57	93	Accepted	Accepted	Accepted	−13.62	0.13
APL9	0.523	4.922	131	Accepted	Accepted	Accepted	−6.21	0.17
APL10	0.515	4.116	99	Accepted	Accepted	Accepted	−9.14	0.20
APL11	0.528	4.585	124	Accepted	Accepted	Accepted	−12.72	0.17
APL12	0.527	3.867	96	Accepted	Accepted	Accepted	−9.77	0.16
APL13	0.537	3.965	98	Accepted	Accepted	Accepted	−7.09	0.19
APL14	0.545	4.051	96	Accepted	Accepted	Accepted	−6.98	0.12
APL15	0.662	3.888	99	Accepted	Accepted	Accepted	−4.85	0.09
APL16	0.649	5.432	153	Accepted	Accepted	Accepted	−7.68	0.07
APL17	0.653	5.294	153	Accepted	Accepted	Accepted	−8.15	0.16
APL18	0.674	5.318	153	Accepted	Accepted	Accepted	−8.17	0.17
APL19	0.712	5.29	151	Accepted	Accepted	Accepted	−8.15	0.20
APL20	0.694	4.834	126	Accepted	Accepted	Accepted	−9.71	0.19
APL21	0.683	3.854	100	Accepted	Accepted	Accepted	−6.0	0.08
APL22	0.674	3.718	100	Accepted	Accepted	Accepted	−6.13	0.2
APL23	0.654	4.578	128	Accepted	Accepted	Accepted	−7.28	0.21
APL24	0.63	4.759	127	Accepted	Accepted	Accepted	−7.78	0.22
APL25	0.63	4.822	127	Accepted	Accepted	Accepted	−7.39	0.22
APL26	0.624	4.287	145	Accepted	Accepted	Rejected	−7.94	0.15
APL27	0.531	3.615	80	Accepted	Accepted	Rejected	−6.86	0.15
APL28	0.538	3.54	96	Accepted	Accepted	Rejected	−9.5	0.15
APL29	0.517	3.565	97	Accepted	Accepted	Rejected	−11.19	0.14
APL30	0.556	3.539	64	Accepted	Accepted	Rejected	−8.25	0.16
APL31	0.552	3.073	54	Accepted	Accepted	Rejected	−8.57	0.16
APL32	0.675	3.983	80	Accepted	Accepted	Rejected	−5.09	0.09
APL33	0.456	3.767	80	Accepted	Accepted	Rejected	−8.5	0.12
APL34	0.702	3.758	85	Accepted	Accepted	Rejected	−6.15	0.21
APL35	0.746	3.558	77	Accepted	Accepted	Rejected	−6.73	0.17
APL36	0.705	3.537	77	Accepted	Accepted	Rejected	−5.26	0.17
APL37	0.483	3.135	23	Accepted	Accepted	Rejected	−9.09	0.19
APL38	0.705	3.536	77	Accepted	Accepted	Rejected	−6.54	0.17
APL39	0.504	3.592	80	Accepted	Accepted	Rejected	−4.95	0.16
APL40	0.572	3.005	54	Accepted	Accepted	Rejected	−8.28	0.17
APL41	0.614	3.033	24	Accepted	Accepted	Rejected	−8.18	0.19
APL42	0.572	3.01	54	Accepted	Accepted	Rejected	−8.28	0.17
APL43	0.709	3.578	36	Accepted	Rejected	Rejected	−9.17	0.25
APL44	0.629	3.026	23	Accepted	Accepted	Rejected	−8.5	0.2
APL45	0.436	4.166	108	Accepted	Accepted	Accepted	−8.88	0.33
APL46	0.534	4.423	124	Accepted	Accepted	Accepted	−8.72	0.09
APL47	0.531	4.048	100	Accepted	Accepted	Accepted	−5.87	0.14
APL48	0.522	3.817	98	Accepted	Accepted	Accepted	−6.89	0.17
APL49	0.516	3.855	95	Accepted	Accepted	Accepted	−9.87	0.23
APL50	0.522	3.837	95	Accepted	Accepted	Accepted	−4.33	0.29
APL51	0.518	3.988	95	Accepted	Accepted	Accepted	−7.1	0.24
APL52	0.551	3.345	92	Accepted	Accepted	Accepted	−4.14	0.32
APL53	0.551	3.425	92	Accepted	Accepted	Accepted	−4.14	0.32
APL54	0.535	3.547	95	Accepted	Accepted	Accepted	−3.78	0.31
APL55	0.569	3.392	92	Accepted	Accepted	Accepted	−5.22	0.30
APL56	0.569	3.387	92	Accepted	Accepted	Accepted	−5.22	0.30
APL57	0.528	3.545	94	Accepted	Accepted	Accepted	−10.13	0.23
APL58	0.531	3.543	95	Accepted	Accepted	Accepted	−5.2	0.30
APL59	0.569	3.52	92	Accepted	Accepted	Accepted	−5.22	0.31
APL60	0.569	3.476	92	Accepted	Accepted	Accepted	−5.22	0.31
APL61	0.548	3.607	92	Accepted	Accepted	Accepted	−5.59	0.29
APL62	0.528	3.614	95	Accepted	Accepted	Accepted	−5.17	0.29
APL63	0.518	3.625	95	Accepted	Accepted	Accepted	−12.46	0.15
APL64	0.502	3.522	94	Accepted	Accepted	Accepted	−5.37	0.16
APL65	0.503	3.881	94	Accepted	Accepted	Accepted	−4.16	0.27
APL66	0.496	4.001	128	Accepted	Accepted	Accepted	−6.27	0.17
APL67	0.502	3.536	113	Accepted	Rejected	Accepted	−4.76	0.13
APL68	0.518	3.625	95	Accepted	Accepted	Accepted	−12.46	0.15
APL69	0.395	3.526	98	Accepted	Accepted	Accepted	−5.32	0.15
APL70	0.425	3.672	104	Accepted	Accepted	Rejected	−8.14	0.13
APL71	0.298	3.608	99	Accepted	Accepted	Accepted	−10.20	0.14
APL72	0.457	3.632	105	Accepted	Rejected	Rejected	−8.78	0.1
APL73	0.53	3.439	95	Accepted	Rejected	Accepted	−5.17	0.15
APL74	0.365	3.71	106	Accepted	Accepted	Rejected	−9.66	0.11
APL75	0.734	3.589	86	Accepted	Accepted	Accepted	−3.65	0.29
APL76	0.705	3.626	89	Accepted	Accepted	Accepted	−5.35	0.26
APL77	0.554	3.554	76	Accepted	Accepted	Accepted	−13.66	0.23
APL78	0.734	3.671	76	Accepted	Accepted	Accepted	−11.74	0.21
APL79	0.674	3.678	86	Accepted	Rejected	Accepted	−10.26	0.16
APL80	0.668	4.296	136	Accepted	Rejected	Accepted	−8.27	0.15
APL	0.538	3.54	96	Accepted	Accepted	Accepted	−9.5	0.15

QED, quantitative estimate of druglikeness; Synth, synthetic accessibility score; Fsp3, The number of sp3 hybridized carbons/total carbon count; MCE-18, medicinal chemistry evolution in 2018; GT, golden triangle; DL, drug likeness; DS, drug score.

### 3.4 Prediction of DS and DL score

The definitions of DL and DS have been established according to certain physicochemical properties of the known drug compounds and how they influence the molecular behavior *in-vitro*. The DL and DS scores predict the properties, such as solubility, permeability, metabolic stability, and transporter effects, of the drug candidates. The DL score of the compounds may provide information about their safety and efficacy. The DS is a comprehensive metric that compiles properties including druglikeness, cLogP, logS, MW, and toxicity concerns into a single number. This value may be used to assess the overall possibility of an unknown compound meeting the criteria for becoming a drug (Bickerton et al., 2012; Lagu et al., 2022). Among all analogues, APL75 has a higher DL score, followed by APL54 with scores of −3.65 and −3.78, respectively, compared to APL (−9.5). On the other hand, APL45 (0.33) has a higher DS, followed by ADL54, ADL59, and ADL60 with a score of 0.31, which is superior to APL (0.15). The predicted DL and DS scores are shown in Table 1.

### 3.5 Prediction of pharmacokinetic (ADME) properties

Pharmacokinetic parameters play a crucial role in drug development, and they provide valuable information on the absorption, distribution, metabolism, and excretion of a drug inside the body. We have calculated the pharmacokinetic parameters, including absorption (caco-2, MDCK, and HIA), distribution (BBB, PPB, and VD), metabolism (CYP3A4), and excretion (CL and T_1/2_), for the designed analogues. The scores for these parameters are tabulated in Table 2. The human colon adenocarcinoma cell line (Caco-2) is a widely used *in-vitro* method for predicting the intestinal permeability of drugs and assessing their potential for oral absorption. Therefore, the assessment of Caco-2 cell permeability has emerged as a crucial criterion in determining the suitability of a therapeutic molecule. Results reflecting the caco-2 score of analogues APL2, APL3, APL5, APL8, APL9, APL12, APL14, APL18, APL19, APL27-30, APL32-38, APL40-53, APL55-69, APL71-73, APL75, and APL77-80 found more than −5.15, which may be predicted as proper *in-vivo* drug permeability. An excellent MDCK score suggests that all analogues have the ability to permeate and transport across the cell. Human intestinal absorption (HIA) scores found in the range between 0 and 0.3 indicate analogues might have good oral bioavailability. The blood-brain barrier (BBB) score for analogues such as APL11, APL24, APL25, and APL37 is less than 0.03, indicating that these analogues might be safe from CNS side effects.

**TABLE 2:** ADME properties of the analogues.

Entry no.	Caco-2	MDCK	HIA	BBB	PPB (%)	VD	CYP3A4	CL	T_1/2_
APL1	−5.256	Ex	0.008	0.49	94.20	0.456	sub	1.383	0.172
APL2	−5.062	Ex	0.005	0.832	85.95	0.978	sub	4.292	0.175
APL3	−5.062	Ex	0.005	0.832	85.95	0.978	sub	4.292	0.175
APL4	−5.203	Ex	0.006	0.688	81.69	2.279	sub	4.827	0.062
APL5	−5.107	Ex	0.007	0.609	93.86	1.403	sub	5.058	0.077
APL6	−5.265	Ex	0.008	0.487	74.68	2.65	sub	6.753	0.107
APL7	−5.282	Ex	0.005	0.355	94.48	0.923	sub	3.63	0.167
APL8	−5.04	Ex	0.006	0.474	90.46	1.488	sub	6.308	0.131
APL9	−5.122	Ex	0.016	0.875	78.30	0.881	sub	8.422	0.229
APL10	−5.15	Ex	0.018	0.577	72.81	0.983	sub	4.9	0.29
APL11	−5.424	Ex	0.037	0.226	62.86	0.66	sub	7.038	0.33
APL12	−4.747	Ex	0.011	0.634	83.59	1.033	sub	6.72	0.256
APL13	−5.204	Ex	0.023	0.963	89.47	1.645	sub	6.922	0.157
APL14	−5.008	Ex	0.018	0.481	93.25	0.656	sub	6.932	0.288
APL15	−5.626	Ex	0.015	0.401	70.40	0.913	sub	5.814	0.208
APL16	−5.489	Ex	0.014	0.506	80.29	0.746	sub	7.259	0.202
APL17	−5.179	Ex	0.011	0.827	85.49	1.01	sub	7.628	0.162
APL18	−5.126	Ex	0.013	0.911	75.20	1.025	sub	7.527	0.211
APL19	−5.128	Ex	0.015	0.837	73.06	0.981	sub	7.129	0.177
APL20	−5.288	Ex	0.04	0.967	71.14	1.15	sub	3.799	0.305
APL21	−5.682	Ex	0.01	0.711	59.80	0.942	sub	6.049	0.184
APL22	−5.628	Ex	0.013	0.932	77.97	0.937	sub	6.348	0.227
APL23	−5.456	Ex	0.014	0.417	60.99	0.926	sub	5.195	0.36
APL24	−5.408	Ex	0.015	0.191	46.90	0.795	sub	4.049	0.438
APL25	−5.377	Ex	0.018	0.200	44.78	0.853	sub	3.74	0.363
APL26	−5.407	Ex	0.011	0.500	95.81	0.922	sub	5.03	0.18
APL27	−5.088	Ex	0.019	0.941	91.67	1.021	sub	7.157	0.189
APL28	−5.091	Ex	0.01	0.931	93.53	1.203	sub	6.294	0.123
APL29	−5.077	Ex	0.008	0.855	94.63	1.101	sub	6.354	0.102
APL30	−5.095	Ex	0.011	0.927	93.04	1.188	sub	6.142	0.141
APL31	−5.188	Ex	0.018	0.962	91.18	1.126	sub	6.782	0.183
APL32	−5.068	Ex	0.011	0.651	94.54	0.484	sub	7.466	0.039
APL33	−5.026	Ex	0.018	0.708	95.25	0.953	sub	7.568	0.129
APL34	−5.039	Ex	0.032	0.719	65.66	1.408	sub	6.595	0.27
APL35	−5.008	Ex	0.006	0.949	90.92	0.779	sub	7.576	0.058
APL36	−4.912	Ex	0.006	0.743	90.45	0.708	sub	6.763	0.093
APL37	−4.933	Ex	0.013	0.105	81.27	0.449	inh	4.584	0.623
APL38	−4.96	Ex	0.006	0.909	90.75	0.75	sub	7.999	0.071
APL39	−5.158	Ex	0.022	0.913	89.81	0.915	sub	7.826	0.213
APL40	−4.934	Ex	0.015	0.99	84.34	0.943	sub	6.335	0.154
APL41	−4.81	Ex	0.009	0.518	79.78	0.486	sub	3.739	0.696
APL42	−4.92	Ex	0.015	0.99	84.78	0.944	sub	6.315	0.16
APL43	−4.723	Ex	0.005	0.448	97.92	1.255	sub	8.788	0.062
APL44	−4.913	Ex	0.006	0.301	75.59	0.511	sub	3.418	0.697
APL45	−5.028	Ex	0.016	0.965	86.72	0.524	sub	5.619	0.16
APL46	−4.985	Ex	0.013	0.909	90.23	1.117	sub	4.822	0.117
APL47	−5.063	Ex	0.017	0.876	49.73	1.092	sub	2.724	0.485
APL48	−5.118	Ex	0.013	0.978	76.24	1.817	sub	2.933	0.112
APL49	−4.876	Ex	0.013	0.308	94.54	1.134	sub	7.069	0.221
APL50	−4.922	Ex	0.013	0.989	87.40	2.027	sub	5.135	0.107
APL51	−4.811	Ex	0.023	0.956	88.56	1.953	sub	5.899	0.137
APL52	−4.977	Ex	0.006	0.968	93.03	1.918	sub	6.612	0.14
APL53	−5.034	Ex	0.006	0.97	92.29	2.239	inh	6.627	0.135
APL54	−5.31	Ex	0.01	0.409	89.38	1.178	sub	4.904	0.191
APL55	−4.925	Ex	0.005	0.973	89.24	2.215	sub	3.659	0.12
APL56	−4.927	Ex	0.005	0.967	88.90	2.228	sub	3.931	0.132
APL57	−4.886	Ex	0.006	0.992	91.44	2.191	sub	3.83	0.12
APL58	−4.991	Ex	0.008	0.767	92.50	1.998	sub	2.287	0.229
APL59	−4.838	Ex	0.007	0.959	89.48	1.571	sub	4.114	0.108
APL60	−4.806	Ex	0.007	0.974	86.63	1.959	sub	3.154	0.088
APL61	−4.84	Ex	0.007	0.785	94.10	2.293	sub	7.835	0.142
APL62	−4.78	Ex	0.011	0.982	93.67	1.887	sub	4.206	0.106
APL63	−5.083	Ex	0.012	0.985	85.52	2.206	sub	3.45	0.177
APL64	−4.965	Ex	0.007	0.976	89.84	2.131	sub	3.662	0.149
APL65	−5.131	Ex	0.009	0.891	92.44	1.851	sub	7.14	0.156
APL66	−5.096	Ex	0.007	0.991	86.52	1.744	sub	3.119	0.203
APL67	−5.142	Ex	0.005	0.982	95.24	1.747	sub	2.716	0.05
APL68	−5.083	Ex	0.012	0.985	85.52	2.206	sub	3.45	0.177
APL69	−5.061	Ex	0.008	0.919	91.55	2.405	sub	2.836	0.129
APL70	−5.368	Ex	0.009	0.989	94.41	1.962	sub	2.63	0.085
APL71	−4.924	Ex	0.008	0.583	94.17	1.379	sub	3.612	0.133
APL72	−5.138	Ex	0.008	0.510	97.46	4.615	sub	5.005	0.055
APL73	−4.908	Ex	0.011	0.846	95.08	1.87	sub	5.253	0.073
APL74	−5.318	Ex	0.011	0.951	95.00	1.991	sub	1.803	0.094
APL75	−5.099	Ex	0.026	0.972	90.43	0.859	sub	7.002	0.247
APL76	−5.172	Ex	0.016	0.972	91.75	0.882	sub	6.428	0.138
APL77	−4.993	Ex	0.01	0.952	66.95	0.943	sub	7.191	0.439
APL78	−4.983	Ex	0.008	0.986	85.02	0.94	sub	6.26	0.26
APL79	−4.984	Ex	0.004	0.947	93.89	0.917	sub	5.892	0.192
APL80	−4.968	Ex	0.005	0.716	95.23	2.754	inh	8.184	0.105
APL	−5.091	Ex	0.01	0.931	93.53	1.203	sub	6.294	0.123

Caco-2, the human colon adenocarcinoma cell lines; MDCK, Madin−Darby canine kidney cells; HIA, human intestinal absorption; PPB, plasma protein binding; BBB, blood–brain barrier; VD, volume distribution; Fu, the fraction unbound in plasms; Ex, Excellent; sub, substrate for human cytochrome P450 (CYP3A4); CL, the clearance of a drug; T_1/2_, the half-life of a drug.

The designed analogues, including APL2-4, APL6, APL9-13, APL15-25, APL34, APL37, APL39-42, APL44, APL45, APL47, APL48, APL50, APL51, APL53, APL54-56, APL59, APL60, APL64, APL66, APL68, APL77, and APL78, exhibited plasma protein binding (PPB) of less than 90%. Therefore, these compounds may have proper PPB, indicating that they can distribute easily throughout the body. The volume of distribution (VD) of all analogues shows a score in the range between 0.04 and 20, which means that these analogues may have a proper distribution amount in body fluid and an uptake amount in tissues. Cytochrome P450 (CYT P450) is a group of isozymes that plays a crucial role in phase-I and phase-II drug metabolism. All analogues show a higher substrate score and a lower inhibitor score for CYP3A4, with the exception of APL80, which means they might be easily metabolized in the body. Analyses have found that the clearance (CL) score of analogues such as APL5-6, APL8, APL9, APL11-14, APL15-19, APL21-23, APL26, APL27-36, APL42, APL43, APL45, APL49-53, APL61, APL65, APL72, APL73, and APL75-80 is greater than 5. The clarity of the drug candidates indicates the dosing frequency of a drug. Half-life (T_1/2_) score of all analogues found in the range from 0 to 0.3 with some exception of APL11, APL20, APL23-25, APL37, APL41, APL44, APL47, and APL77, which indicates proper clearance from the body.

### 3.6 Prediction of the toxicity properties

The toxicological properties of analogues, including drug-induced liver injury (DILI), mutagenicity (Ames test), acute oral toxicity in rats (ROA), binding of the molecule with the ligand-binding domain (LBD) of the androgen receptor (NR-AR-LBD), and carcinogenicity (Carc.), were calculated, and their scores are shown in Table 3. All designed analogues had identical human hepatotoxicity (H-HT) scores to APL, indicating they may have shown harmful effects. Analogue APL29 was predicted to have a safer DILI score, while APL exhibited toxicity (0.99). However, counterparts such as APL32, APL35, APL36, and APL38 predicted mild toxicity levels ranging from 0.3 to 0.7. However, the prediction of a safer mutagenic score for all analogues indicates that they are unlikely to induce mutagenesis. However, there are several exceptions, including APL15, APL20, APL32, APL33, APL35, APL36, APL38, APL41, APL44, APL46, APL49, APL51, APL61, and APL80.

**TABLE 3:** Toxicity screening of analogues.

Entry no.	H-HT	DILI	Ames	ROA	Carc.	NR-AR	NR-AR-LBD
APL1	0.984	0.996	0.02	0.939	0.877	0.375	0.243
APL2	0.969	0.988	0.042	0.893	0.862	0.319	0.13
APL3	0.969	0.988	0.042	0.893	0.862	0.319	0.13
APL4	0.985	0.99	0.026	0.911	0.597	0.015	0.369
APL5	0.987	0.994	0.022	0.739	0.906	0.047	0.217
APL6	0.984	0.983	0.02	0.947	0.712	0.056	0.062
APL7	0.979	0.995	0.042	0.938	0.875	0.472	0.114
APL8	0.984	0.989	0.045	0.911	0.898	0.09	0.527
APL9	0.981	0.984	0.043	0.634	0.893	0.012	0.007
APL10	0.982	0.993	0.055	0.605	0.892	0.034	0.04
APL11	0.964	0.997	0.061	0.644	0.754	0.015	0.004
APL12	0.966	0.994	0.083	0.766	0.605	0.01	0.181
APL13	0.971	0.989	0.087	0.633	0.878	0.026	0.048
APL14	0.975	0.994	0.043	0.238	0.842	0.011	0.022
APL15	0.99	0.986	0.358	0.943	0.983	0.329	0.014
APL16	0.989	0.98	0.25	0.73	0.95	0.004	0.004
APL17	0.936	0.978	0.029	0.607	0.931	0.017	0.013
APL18	0.933	0.98	0.027	0.645	0.934	0.011	0.012
APL19	0.939	0.981	0.034	0.609	0.936	0.009	0.015
APL20	0.963	0.986	0.372	0.925	0.972	0.009	0.078
APL21	0.986	0.982	0.247	0.836	0.969	0.206	0.02
APL22	0.982	0.984	0.046	0.944	0.945	0.27	0.01
APL23	0.985	0.978	0.052	0.864	0.896	0.008	0.005
APL24	0.982	0.984	0.298	0.844	0.9	0.002	0.008
APL25	0.973	0.981	0.202	0.817	0.877	0.005	0.005
APL26	0.958	0.987	0.07	0.583	0.838	0.032	0.27
APL27	0.97	0.99	0.026	0.754	0.852	0.027	0.087
APL28	0.975	0.991	0.039	0.843	0.841	0.037	0.285
APL29	0.973	0.99	0.038	0.838	0.84	0.036	0.262
APL30	0.976	0.992	0.046	0.828	0.851	0.039	0.406
APL31	0.976	0.989	0.03	0.778	0.786	0.036	0.05
APL32	0.9	0.48	0.397	0.649	0.214	0.262	0.603
APL33	0.964	0.774	0.52	0.31	0.069	0.137	0.425
APL34	0.968	0.935	0.021	0.118	0.107	0.001	0.002
APL35	0.931	0.485	0.604	0.787	0.431	0.121	0.661
APL36	0.931	0.485	0.604	0.787	0.431	0.121	0.661
APL37	0.978	0.982	0.718	0.288	0.609	0.072	0.587
APL38	0.967	0.334	0.463	0.857	0.517	0.139	0.417
APL39	0.969	0.986	0.245	0.677	0.875	0.013	0.027
APL40	0.969	0.989	0.056	0.869	0.173	0.409	0.041
APL41	0.977	0.957	0.423	0.583	0.168	0.112	0.476
APL42	0.97	0.989	0.061	0.87	0.17	0.409	0.043
APL43	0.978	0.251	0.088	0.758	0.175	0.017	0.565
APL44	0.982	0.977	0.658	0.419	0.124	0.086	0.187
APL45	0.976	0.99	0.228	0.194	0.932	0.034	0.009
APL46	0.98	0.995	0.671	0.517	0.924	0.008	0.003
APL47	0.987	0.99	0.149	0.318	0.963	0.009	0.009
APL48	0.95	0.986	0.068	0.673	0.9	0.097	0.063
APL49	0.979	0.99	0.491	0.975	0.861	0.021	0.318
APL50	0.973	0.985	0.028	0.972	0.439	0.027	0.008
APL51	0.971	0.991	0.044	0.993	0.438	0.035	0.011
APL52	0.887	0.982	0.031	0.839	0.681	0.023	0.005
APL53	0.903	0.986	0.081	0.82	0.524	0.034	0.005
APL54	0.926	0.99	0.03	0.892	0.692	0.037	0.008
APL55	0.911	0.992	0.037	0.849	0.518	0.024	0.006
APL56	0.916	0.991	0.034	0.867	0.615	0.025	0.005
APL57	0.939	0.986	0.032	0.637	0.881	0.116	0.011
APL58	0.915	0.992	0.046	0.547	0.721	0.037	0.035
APL59	0.979	0.985	0.046	0.878	0.884	0.068	0.038
APL60	0.952	0.993	0.038	0.596	0.744	0.029	0.007
APL61	0.951	0.989	0.433	0.964	0.772	0.018	0.008
APL62	0.968	0.994	0.049	0.68	0.742	0.035	0.013
APL63	0.951	0.993	0.039	0.804	0.803	0.046	0.064
APL64	0.939	0.99	0.041	0.677	0.785	0.042	0.021
APL65	0.965	0.987	0.02	0.963	0.326	0.021	0.005
APL66	0.907	0.987	0.024	0.784	0.854	0.121	0.019
APL67	0.925	0.986	0.033	0.866	0.881	0.028	0.032
APL68	0.951	0.993	0.039	0.804	0.803	0.046	0.064
APL69	0.929	0.99	0.025	0.81	0.859	0.049	0.017
APL70	0.945	0.996	0.028	0.765	0.794	0.015	0.017
APL71	0.938	0.989	0.119	0.699	0.912	0.3	0.09
APL72	0.984	0.992	0.024	0.95	0.809	0.018	0.065
APL73	0.949	0.989	0.023	0.864	0.782	0.155	0.019
APL74	0.956	0.993	0.108	0.795	0.885	0.022	0.037
APL75	0.989	0.989	0.069	0.9	0.956	0.017	0.063
APL76	0.978	0.991	0.024	0.383	0.755	0.007	0.023
APL77	0.95	0.987	0.207	0.811	0.863	0.156	0.12
APL78	0.939	0.984	0.045	0.832	0.916	0.085	0.043
APL79	0.951	0.988	0.057	0.343	0.86	0.019	0.725
APL80	0.972	0.992	0.317	0.981	0.863	0.005	0.176
APL	0.975	0.991	0.039	0.843	0.841	0.037	0.285

H-HT, the human hepatotoxicity; DILI, drug-induced liver injury; Ames, Test for mutagenicity; ROA, rat oral acute toxicity; NR-AR, androgen receptor - a nuclear hormone receptor; NR-AR-LBD, molecule bind with LBD, of androgen receptor; Carc., carcinogenicity.

Analogues APL14, APL34, APL45, and APL77 exhibited ROA prediction scores within a safer range (0–0.3), a crucial safety characteristic for potential drug candidates. In contrast, ROA score of APL found in toxic range. APL9-11, APL13, APL17-19, APL26, APL32-33, APL39, APL41, APL44, APL46-48, APL53, APL57, APL58, APL60, APL62, APL64, APL71, and APL76 exhibited lower levels of hazards compared to APL. The carcinogenic nature of analogues is a significant concern due to their potent impact on health and their ability to harm the genome or disturb cellular metabolism. The NR-AR receptor plays a vital function in androgen receptor-dependent PC and other disorders connected to androgens. Researchers determined that the analogues APL4-6, APL8, APL9-16, APL27-39, APL41, and APL43-80 have a lower NR-AR score, suggesting their potential non-toxicity to the AR.

### 3.7 Molecular docking study

Our objective was to examine the possible interaction between newly designed APL analogues and the protein. We acquired the 3D crystallographic structure of the protein from the protein data bank (*PDB ID: 5T8E*). The ADV program successfully aligned the protein with the ligands (Supplementary Table S1), resulting in uniform grid box dimensions that enhanced the understanding of the inhibitors' binding affinities. The designed ligands exhibited docking scores ranging from −6.2 to −8.5 *Kcal/mol* (Table 4).

**TABLE 4:** Docking score and interactions of the analogues.

Entry no.	Docking score (*Kcal/mol*)	Interactions	
		H-binding	Other
APL1	-	NHB	NHB
APL2	−6.9	ARG752, THR755, TRP751	ARG752, GLU681, ALA748, PRO801, PHE804, PRO801
APL3	−6.8	ARG752, THR755	GLU681, ALA748, ARG752, LEU805, PRO801
APL4	−6.9	ARG752, THR755	GLY683, PRO801, VAL684, LEU805, ARG752
APL5	−6.6	ARG752, THR755, GLN802, GLU678	GLU678, PRO682, GLY683, VAL684, TRP751, ARG752
APL6	−6.8	VAL685, GLN711, ARG752, THR755, PRO682	GLU681, PRO682, GLY683, TRP751, PHE754, ARG752
APL7	−8.1	GLN711, ARG752, GLU678, VAL684	PRO682, GLU681, GLY683
APL8	−8.4	GLN711, ARG752, PRO682, TRP751	VAL684, GLU681, GLY683
APL9	−7.1	ARG752, THR755, TRP751, ILE799	GLU681, ALA748, PRO801, ARG752, TRP751, PHE804
APL10	−7.2	ARG752, THR755, TRP751, PRO682	GLU681, ALA748, PRO748, ARG752, TRP751
APL11	-	NHB	NHB
APL12	-	NHB	NHB
APL13	−7.6	ARG752, VAL685, TRP751, GLN711	GLU678, GLU681, PRO682, GLY683, TRP751, ARG752
APL14	−7.1	ARG752, PRO682, TRP751, GLN711	GLU681, ARG752, ALA748, PRO682, TRP751
APL15	−7.6	GLN711, ARG752, THR755, ASN756, GLU678, VAL684	GLU681, PRO682, GLY683, TRP751, VAL684, ARG752
APL16	−7.1	ARG752, TRP751, TRP751	GLU681, ALA748, ARG752, TRP751, PHE804
APL17	−7.6	GLN711, ARG752, THR755, GLU678, VAL684	GLU681, PRO682, GLY683, VAL684, ARG752
APL18	−7.6	ARG752, TRP751	GLU681, ALA748, PRO801, ARG752, LEU805, TRP751, TRP751, PHE804, ARG752
APL19	−7.7	VAL685, GLN711, TRP751, ARG752, PRO682	GLU681, PRO682, GLY683, TRP751, PRO682, ARG752
APL20	−7.0	VAL685, GLN711, ARG752	GLU681, PRO682, GLY683, TRP751, ARG752
APL21	−7.9	GLN711, ARG752, GLU678, VAL684	GLU681, PRO682, GLY683, VAL684, ARG752
APL22	−7.7	GLN711, ARG752, PRO682, TRP751, GLU678	GLU681, PRO682, GLY683, TRP751, ARG752
APL23	−7.4	GLN711, ARG752, THR755, ASN756, PRO682	GLU681, PRO682, GLY683, ARG752
APL24	−6.7	GLN711, ARG752, ASN756, VAL684	GLU681, PRO682, GLY683, VAL684, ARG752
APL25	−7.6	ARG752, THR755, TRP751	GLU681, ALA748, ARG752, PHE804
APL26	−7.8	GLN711, ARG752, PRO682	GLU681, PRO682, GLY683, TRP751, VAL684, ARG752
APL27	−8.2	ARG752, THR755, GLU678, PRO682, GLN711	GLU681, PRO682, GLY683, TRP751, VAL684, ARG752
APL28	-	NHB	NHB
APL29	−7.3	ARG752, THR755, VAL685, PRO682, GLN711	GLU681, PRO682, GLY683, THR755, TRP751, ARG752
APL30	−8.2	ARG752, THR755, VAL685, PRO682, GLN711	GLU681, PRO682, GLY683, THR755, TRP751, ARG752
APL31	-	NHB	NHB
APL32	-	NHB	NHB
APL33	−7.4	ARG752, THR755, VAL684, GLN711, GLU678	GLU678, GLU681, PRO682, ARG752, GLU678, TRP751, ALA748, VAL684
APL34	-	NHB	NHB
APL35	−7.1	GLN711, ARG752, ASN756, TYR763, TRP751	GLY683, GLN711, ARG752, VAL684, PRO682, VAL685, ALA748
APL36	-	NHB	NHB
APL37	-	NHB	NHB
APL38	-	NHB	NHB
APL39	-	NHB	NHB
APL40	-	NHB	NHB
APL41	-	NHB	NHB
APL42	−8.5	GLN711, ARG752, GLU678, PRO682, VAL684	GLU681, PRO682, GLY683, VAL684, ARG752
APL43	−6.6	GLN711, ARG752, TRP751, THR755	GLU681, GLN711, ARG752, ALA748, LEU805, PRO682
APL44	-	NHB	NHB
APL45	-	NHB	NHB
APL46	-	NHB	NHB
APL47	−8.3	GLN711, ARG752, TRP751	GLU681, ALA748, TRP751, ARG752
APL48	-	NHB	NHB
APL49	-	NHB	NHB
APL50	-	NHB	NHB
APL51	−7.3	GLN711, ARG752, TRP751, THR755, PRO682, GLY683	GLU681, ALA748, ARG752, PRO801, LEU805
APL52	-	NHB	NHB
APL53	-	NHB	NHB
APL54	−7.7	GLU678, ARG752, TRP751, PRO682	GLU678, GLU681, ALA748, ARG752, TRP751, PHE804
APL55	-	NHB	NHB
APL56	-	NHB	NHB
APL57	-	NHB	NHB
APL58	−7.0	ARG752, TRP751, THR755	GLU681, ARG752, PRO682, GLY683, ALA748, VAL684
APL59	-	NHB	NHB
APL60	-	NHB	NHB
APL61	-	NHB	NHB
APL62	−6.6	GLN711, ARG752, VAL685, GLY683, PHE764	GLU681, PRO682, VAL684, ARG752, VAL685, VAL684
APL63	-	NHB	NHB
APL64	−7.5	GLN711, ARG752, TRP751, GLU678	GLU678, GLU681, ALA748, ARG752, TRP751, PHE804
APL65	-	NHB	NHB
APL66	-	NHB	NHB
APL67	−6.7	TRP751, ARG752, GLU678	GLU681, ALA748, GLU678, PRO682, ARG752, TRP751, PHE804
APL68	-	NHB	NHB
APL69	-	NHB	NHB
APL70	−8.3	GLN711, ARG752, VAL685	GLU678, PRO682, TRP751, ALA748, VAL715, LEU744, LYS808, TRP718, GLY683
APL71	-	NHB	NHB
APL72	−7.6	GLN711, ARG752, TRP751	PRO682, TRP751, ALA748, GLU681, GLY683
APL73	-	NHB	NHB
APL74	-	NHB	NHB
APL75	-	NHB	NHB
APL76	−7.2	GLN711, ARG752, VAL685. ASN756, PRO682	ARG752, GLU681, TRP751
APL77	−6.2	GLN711, ARG752, VAL685. TRP751, VAL684, PRO682	TRP751, LEU805, PHE804, PRO801, VAL684, GLU681, PRO682, ARG752, GLY683
APL78	−7.4	GLN711, ARG752, VAL685	PRO685, GLU681, ARG752, GLY683, TRP751, PRO801, PHE804, LEU805
APL79	−7.9	GLN711, ARG752, VAL684	LEU684, GLY683, PRO682, GLU681, ARG752, GLU678, LEU805
APL80	−8.0	GLN711, ARG752, VAL684. PRO682	TRP751, VAL684, GLY683, PRO682, GLU681, ARG752, GLU678
APL	−7.1	GLN711, ARG752, THR755	GLU681, PRO682, TRP751, ALA748, ARG752

NHB, no hydrogen bonds were seen as being found in apalutamide.

**FIGURE 2:**
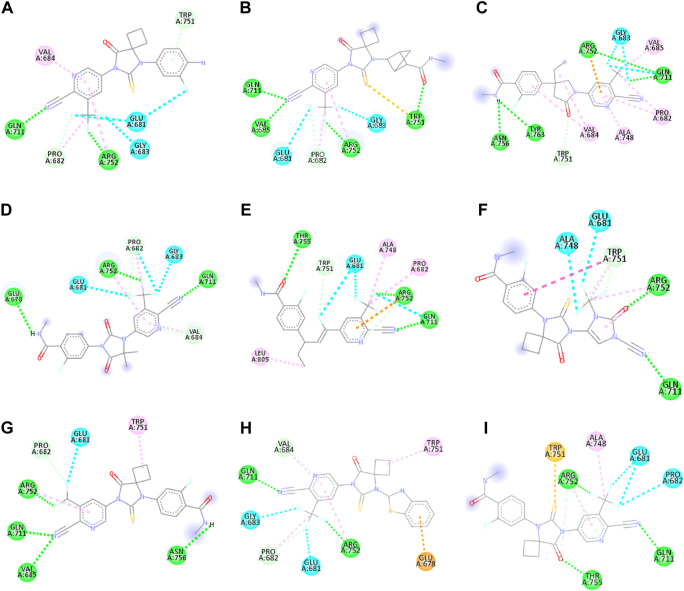
2D interactions of ligand APL8 **(A)**, APL19 **(B)**, APL35 **(C)**, APL 42 **(D)**, APL43 **(E)**, APL47 **(F)**, APL76 **(G)**, APL80 **(H)**, and Apalutamide **(I)**.

**FIGURE 3:**
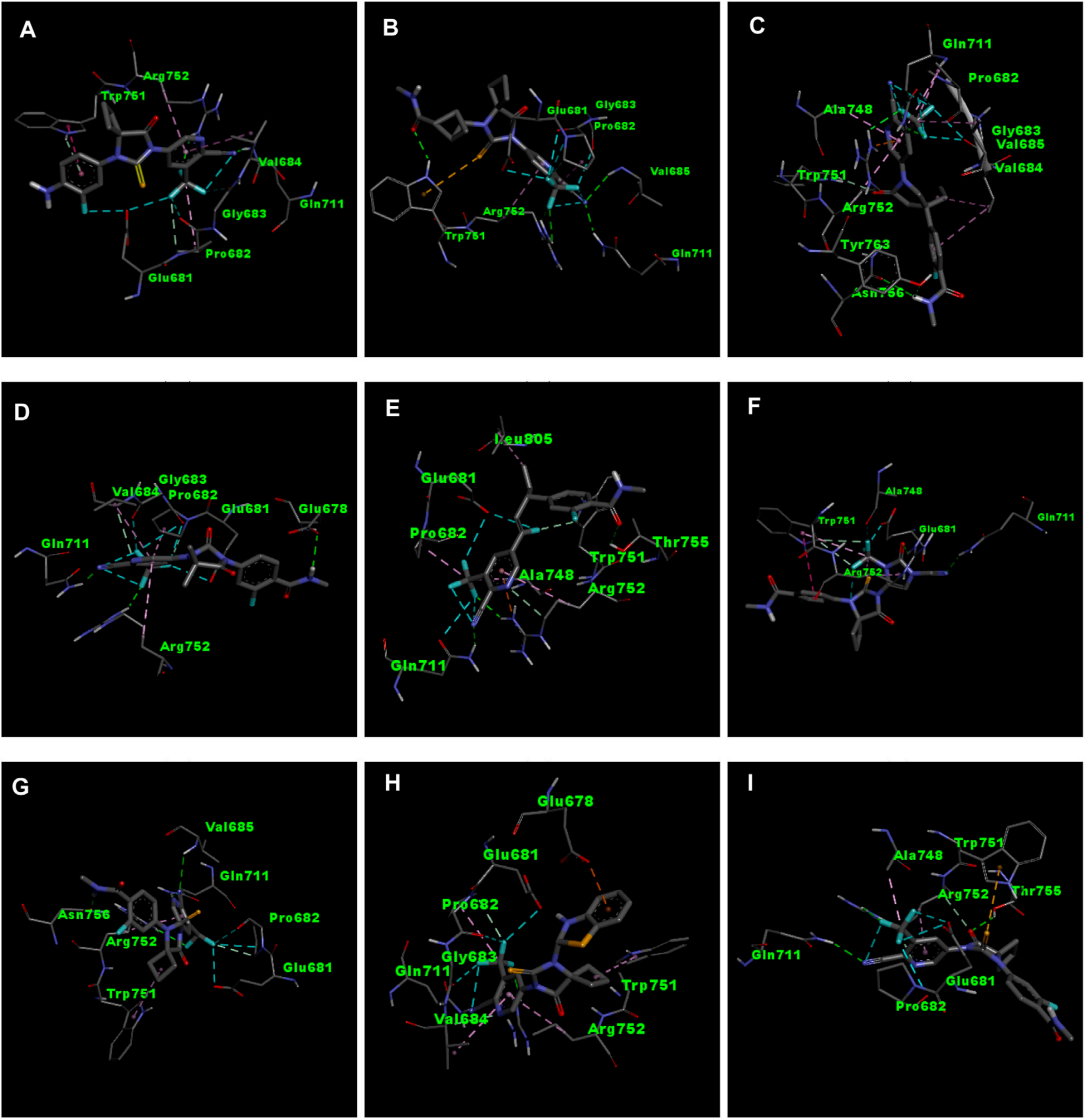
3D interactions of ligand APL8 **(A)**, APL19 **(B)**, APL35 **(C)**, APL 42 **(D)**, APL43 **(E)**, APL47 **(F)**, APL76 **(G)**, APL80 **(H)**, and Apalutamide **(I)**.

APL8 is a bioisostere of APL, where the amide group in the phenyl ring is substituted with an amine group in the phenyl ring. The overall structure of APL8 is comparable to that of APL. Ligand APL8 had the second highest binding affinity score (−8.4 *Kcal/mol*). Residues ARG752 and GLN711 of the target protein form hydrogen bonds with CN and CF_3_ groups in the ligand’s pyridinyl ring in the docked ADV complex. These hydrogen bonds are similar to those found in APL. TRP751 also formed a carbon-hydrogen bond with the phenyl ring of the ligand. On the other hand, it formed an additional hydrogen bond between the GLU678 residue and the N-H group of the methyl amide in the ligand. It was also easy for PRO682 to form strong bonds with the F of the CF_3_ group and the nitrogen of the pyridinyl in the ligand. GLU681 and GLY683 formed a halogen interaction with the F of the CF_3_ group in the ligand. Additionally, ligands formed alkyl (PRO682) and pi-alkyl (VAL684 and ARG752) interactions. Figures 2A, 3A show the 2D and 3D interactions of the ligand APL8, respectively.

APL35 is the bioisostere of APL in which the cyclobutyl-thiohydantoin group is replaced with 3-ethyl-5-oxopyrrolidine, and the resting structure is similar to that of APL. The docking score of APL35 was −7.1 *Kcal/mol*. The APL35 analogue showed four conventional hydrogen bonds with amino acid residues (GLN711, ARG752, ASN756, and TYR763). ARG752 formed a hydrogen bond with fluorine (F) in the trifluoromethyl (CF_3_) group attached to the pyridine ring. GLN711 formed another hydrogen bond with F of CF_3_. The N-H of the amide group formed two hydrogen bonds with ASN756 and TYR763. A carbon-hydrogen bond was also seen between TRP751 and 3-ethyl-5-oxopyrrolidine. ARG752 formed a pi-cation bond with the pyridine ring as well. We identified an alkyl bond between the carbons of CF_3_ and Val685 as well as PRO682. We observed a pi-alkyl bond between the pyridine ring and PRO682, as well as ALA748. There is a halogenic bond form between Gly683 and GLN711 and the F in the CF_3_ group. The 2D and 3D interactions of the ligand APL35 are shown in Figures 2C, 3C, respectively.

A docking study revealed that APL42 shows the highest binding score (−8.5 *Kcal/mol*) among selected analogues. There are cyano (CN) and CF_3_ groups that form hydrogen bonds with GLN711 and ARG752, respectively. There are also hydrogen bonds in APL. Surprisingly, GLU678 forms additional hydrogen bonds with the N-H group of the amide in the ligand. PRO682 shows a carbon-hydrogen bond interaction with the F of CF_3_ groups. The two F atoms of CF_3_ formed halogen bonding with the amino acid residues GLU681, PRO682, and GLY683. Residues VAL684 and ARG752 show pi-alkyl interactions with the pyridinyl group in the ligand. Residue PRO682 also formed an alkyl interaction with F of CF_3_. The 2D and 3D interactions of the ligand APL42 are shown in Figures 2D, 3D, respectively.

APL43 is a bioisostere of APL, where the cyclobutyl-thiohydantoin group is substituted with 1-fluoropent-1-ene. The overall structure of APL43 is comparable to that of APL. The docking score of APL43 was −6.6 *Kcal/mol*. A docking study revealed that APL43 formed three conventional hydrogen bonds with THR755, ARG752, and GLN711. Ligand APL43 formed a hydrogen bond with THR755 through the oxygen attached to the carbon next to the phenyl ring. ARG752 formed a hydrogen bond with one of the F in the CF_3_ group attached to the pyridine ring. The CN group attached to the pyridine ring formed a hydrogen bond with GLN711. The F attached to the double-bonded carbon next to the pyridine ring forms a carbon-hydrogen bond with TRP751. The nitrogen of the pyridine group forms a carbon-hydrogen bond with ARG752. GLU681 and GLN711 form three halogen bonds. GLU681 forms halogen bonds with a double-bonded carbon adjacent to the pyridine ring and one of the F from the CF_3_ group. LEU805 forms an alkyl bond with the last carbon of the ethyl group attached to the carbon next to the phenyl group. The carbon of the CF_3_ group forms alkyl bonds with ALA748 and PRO682. Figures 2E, 3E show the 2D and 3D interactions of the ligand APL43, respectively.

The structure of APL47 is like that of APL, but the 6-cyano-5-(trifluoromethyl)pyridin-3-yl group is changed to 1-cyano-2-oxo-3-(trifluoromethyl)-2,3-dihydro-1H-imidazol-4-yl. The resting structure of APL47 is the same as that of APL. The docking score of APL47 was −8.3 *Kcal/mol*. It was found that residues ARG752 and GLN711 connect with CN and carbonyl groups in 1-cyano-2-oxo-3-(trifluoromethyl)-2,3-dihydro-1H-imidazol, which is the same as APL. TRP751 formed a carbon-hydrogen bond with F of the CF_3_ side chain. Glu681 and ALA748 interacted with the F of CF_3_ through halogen bonds. Hydrophobic interactions formed between the phenyl ring of the ligand and TRP751. Additionally, ARG752 and TRP751 exhibited hydrophobic interactions with the F of CF_3_ and 1,3-dihydro-1H-imidazol, respectively. Figures 2F, 3F show the 2D and 3D interactions of the ligand APL47, respectively.

APL76 is a bioisostere of APL, where the CF_3_ group is substituted with an CF_2_ group in the pyridine ring. The docking score for APL76 was −7.2 *Kcal/mol*. Through ADV, four regular hydrogen bonds were seen between the ligand APL76 and the amino acid residues ASN756, ARG752, GLN711, and VAL685. The hydrogen bonds with VAL685 and GLN711 were via the nitrogen triple bond with the carbon next to the pyridine ring. A hydrogen bond was established between ARG752 and one of the F presents in CF_2_ that was attached to the pyridine ring. ASN756 formed a hydrogen bond with the hydrogen atom attached to the nitrogen atom of the amide group. GLU681 forms a halogen bond with F of the CF_2_. The same F also forms carbon-hydrogen bonds with PRO682. With ARG682, the pyridine ring forms an alkyl bond. TRP751 forms an alkyl bond with the cyclobutene attached to the imidazolidine. Figures 2G, 3G show the 2D and 3D interactions of the ligand APL76, respectively.

APL80 is a bioisostere of APL, where the 6-cyano-5-(trifluoromethyl)pyridin-3-yl group is substituted with a benzothiazoles-2-yl group in the pyridine ring. The docking score for APL80 was −8.0 *Kcal/mol*. According to the study, the CN group of the pyridinyl ring in the ligand formed a hydrogen bond with the GLN711 residue. On the other hand, F of the CF_3_ group also formed a hydrogen bond with the ARG752 residue. Both PRO682 and VAL684 formed a carbon-hydrogen bond with the F of CF_3_ and the nitrogen of the pyridinyl ring in the ligand. Pi-alkyl interactions formed between TRP751 and the cyclobutyl ring of thiohydantoins. GLU681 and GLU683 show interaction with F of CF_3_ through halogenic bonds. A pi-anion bond was formed between the indolyl ring and GLU678. 2D and 3D interactions of the ligand APL80 are shown in Figures 2H, 3H, respectively.

In APL19, the fluoro-phenyl group is replaced with bicyclo [1.1]pentane groups, which is based on the literature (Subbaiah and Meanwell, 2021). The docking score of APL19 was −7.7 *Kcal/mol*. Based on the result of the docking study of APL19, it shows the hydrogen bonding interaction of CN and CF_3_ with GLN711 and F of CF_3_, respectively. Surprisingly, the CN group also forms hydrogen with the VAL685 amino acid residue. On the other hand, the carbonyl group also formed a hydrogen bond with TRP 751. TRP751 also formed a pi-Sulfur interaction with the Sulphur of the thiohydantoin scaffold. GLU681 and GLY683 show halogen bonding with F in CF_3_. The 2D and 3D interactions of the ligand APL19 are shown in Figures 2B, 3B, respectively.

The standard APL shows three conventional hydrogen bonds with the amino acid residues ARG752, THR755, and GLN711. The hydrogen bond with ARG752 was established with one of the F presents in the CF_3_ that was attached to the pyridine ring. The hydrogen bond with GLN711 was via the nitrogen triple bond with the carbon next to the pyridine ring. The hydrogen bond with THR755 was established via the oxygen group attached to the imidazolidine ring. The CF_3_ group’s F formed three halogen bonds with the amino acid residues PRO682 and GLU681. ARG752 forms two carbon-hydrogen bonds, one with the nitrogen of the pyridine ring and the other with the oxygen attached to the imidazolidine ring. The first carbon-hydrogen bond with ARG752 was with the nitrogen of the pyridine ring, and the other was with the oxygen attached to the imidazolidine ring. ARG752 also formed an alkyl bond with the pyridine ring. ALA748 formed another alkyl bond with the carbon of the CF_3_ group. Lastly, there is a pi-Sulfur bond between the THR751 and the sulfur attached to the imidazolidine ring. 2D and 3D interactions of the ligand APL are shown in Figures 2I, 3I, respectively.

In conclusion, ligands APL19, APL35, APL43, APL76, and APL80 show good docking scores of −7.7, −7.1, −6.6, −7.2, and −8.0 *Kcal/mol*, respectively (Table 5). In contrast, APL has a binding affinity score of −7.1 *Kcal/mol*. We found that hydrogen bond interactions in APL involved the two common protein residues (ARG752 and GLN711), which are similar to these ligands. According to the literature, docking studies of APL were carried out with protein (*PDB ID: 5T8E*). APL formed the hydrogen bonding and carbon hydrogen bonding with the ARG752, THR755, and GLN711 protein residues. On the other hand, it shows interaction with ALA748 and ARG752 through halogenic and hydrophobic pi-alkyl interactions, respectively (Ikwu et al., 2020). ARG752 and GLN711 protein residues were the active residues of protein which might be the reason behind the antagonistic activity toward the AR. The QED, MCE-18, docking score, and docking interactions of selected newer APL analogues (APL19, APL35, APL43, APL76, and APL80) are shown in Table 5.

**TABLE 5:** QED, MCE-18, docking score and docking interactions of selected analogues.

Entry no.	QED score	MCE-18 score	Docking score (*Kcal/mol*)	Interactions	
				H-binding	Other
APL19	0.712	151	−7.7	VAL685, GLN711, TRP751, ARG752, PRO682	GLU681, PRO682, GLY683, TRP751, PRO682, ARG752
APL35	0.746	77	−7.1	GLN711, ARG752, ASN756, TYR763, TRP751	GLY683, GLN711, ARG752, VAL684, PRO682, VAL685, ALA748
APL43	0.709	36	−6.6	GLN711, ARG752, TRP751, THR755	GLU681, GLN711, ARG752, ALA748, LEU805, PRO682
APL76	0.705	89	−7.2	GLN711, ARG752, VAL685. ASN756, PRO682	ARG752, GLU681, TRP751
APL80	0.668	136	−8.0	GLN711, ARG752, VAL684. PRO682	TRP751, VAL684, GLY683, PRO682, GLU681, ARG752, GLU678
APL	0.538	96	−7.1	GLN711, ARG752, THR755	GLU681, PRO682, TRP751, ALA748, ARG752

### 3.8 Molecular dynamics simulation

The MD simulation was performed to examine the dynamic behavior of atoms and molecules. It is a methodology and collection of algorithms that calculate and predict the stability of compounds. It is a very effective independent method for accurately capturing molecular and atomistic-level changes. This mechanism is very important for studying how ligand molecules interact with proteins to understand the stability of protein-ligand complexes. Structure-based drug design, which uses standard methods such as molecular docking and virtual screening, has yielded a selection of potential medications in the field of bioscience. The MD simulation is crucial for comprehending the ligand’s dynamic behavior and its stability in relation to the protein. We used the simulation interaction diagram (SID) to examine the MD simulation trajectories of a 100 nS SPC water model-based simulation. This allowed us to get insights into the deviation, fluctuation, and intermolecular interaction occurring throughout the simulation. The MD simulations for ligands APL19, APL35, APL43, APL76, and APL80 were carried out. The MD simulation of ligand APL43 and APL80 were found stable. MD runs for the other molecules (APL19, APL35, and APL76) did not fall within the desired range.

#### 3.8.1 RMSD and RMSF

**FIGURE 4:**
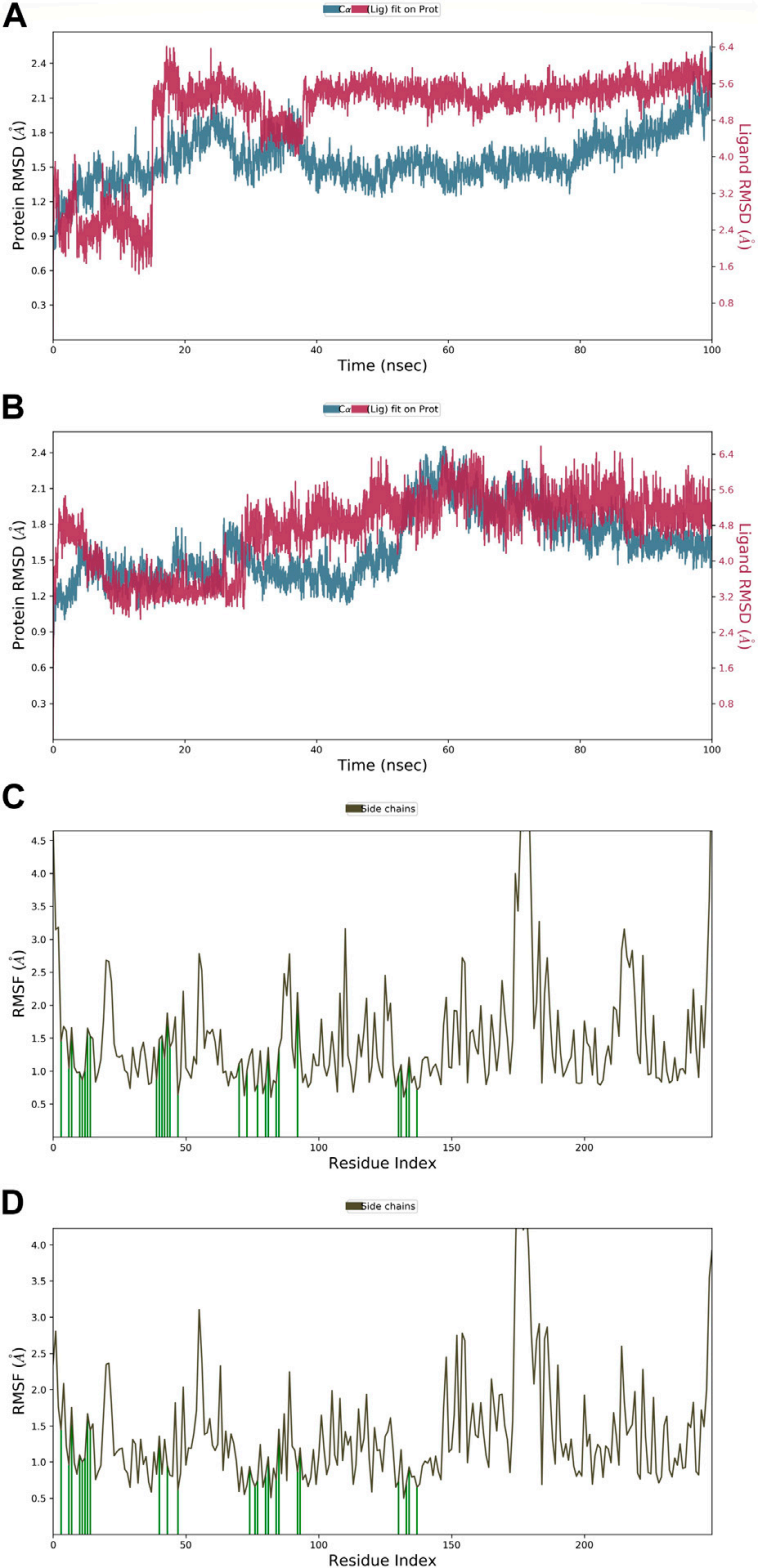
The root mean square deviation (RMSD) of the protein relative to the starting complexes during the 100 nS MD trajectory for APL43 **(A)** and APL80 **(B)**; The root mean square fluctuation (RMSF) of the protein during 100 ns MD, representing local changes along the protein chain for APL43 **(C)** and APL80 **(D)**.

The root mean square deviation (RMSD) quantifies the average displacement of a chosen set of atoms in a certain frame compared to a reference frame. The calculation is performed for every frame in the trajectory. The RMSD value was used to calculate the deviation in the protein’s backbone (C and N) during the 100 nS simulative period. Throughout the MD run, very slight or minute fluctuations in RMSD values were observed as compared to the protein backbone. In the case of a protein in a complex with a ligand (APL43), the backbone RMSD initially fluctuated from 0 to 0.7 Å in 0.50 nS, and the ligand fluctuated 0.8 Å (Figure 4A). We saw that when the protein was in a complex with the ligand (APL80), the protein RMSD changed from 0 to 0.8 Å in 0.50 nS, while the ligand’s changed by 1.1 Å (Figure 4B). The overall RMSD is satisfactory for both complexes. After the initial fluctuation, the complexes throughout the MD run were found stable. The androgen receptor modulator protein in complex with APL43 shows an average RMSD of 1.57 Å, while the ligand shows 2.03 Å at 100 nS. While the androgen receptor modulator protein in complex with APL80 shows an average RMSD of 1.61 Å, the ligand shows 0.75 Å at 100 nS. Initially, we observed a lower RMSD deviation (average RMSD) from 0 to 15 nS; we noticed a slight fluctuation for two frames, followed by stable complexes (APL43) from 15 to 40 nS. After that, we observed a stable RMSD value with minute deviations from 40 to 100 nS. Conversely, we observed a lower RMSD deviation (average RMSD) for APL80 from 0 to 30 nS, a slight fluctuation for three frames, and stable complexes (APL80) from 30 to 60 nS. After that, we observed a stable RMSD value with minute deviations from 60 to 100 nS. This means that both complexes are completely stable for the 100 nS simulation.

Later, the root mean square fluctuation (or RMSF) analysis gives the complex fluctuation with time evolution against amino acid residues. Figures 4C, D show the protein-RMSF and protein-ligand contacts for the complete simulation. We showed how the androgen receptor modulator works with APL43 and APL80 by focusing on how their proteins interact with ligands during 100 nS simulation. On the protein-RMSF plot, peaks indicate areas of the protein that fluctuate the most during the simulation. Typically, we will observe that the tails (N- and C-terminal) fluctuate more than any other part of the protein. Secondary structure elements like alpha helices and beta strands are usually more rigid than the unstructured part of the protein and thus fluctuate less than the loop regions. Green-colored vertical bars mark protein residues that interact with the ligand. In proteins, some amino acid residues have fluctuated (Figures 4C, D). The rest of the amino acid residues have shown a significantly lower level of fluctuations. During the complete 100 nS simulation, ligands APL43 and APL80 showed interaction with lower fluctuation due to the formation of favorable interactions with different amino acid residues. Furthermore, the overall observed fluctuation is very low, providing valuable information for future studies against proteins using both ligands, APL43 and APL80. In addition, the protein molecule is stiffer because of its H-bonds, pi-pi stacking, and secondary structure elements. In both conditions, the fluctuation shown in Figures 4C, D (for ligand APL43 and APL80) is found below 2 Å, indicating promising results.

#### 3.8.2 Intermolecular interactions

**FIGURE 5:**
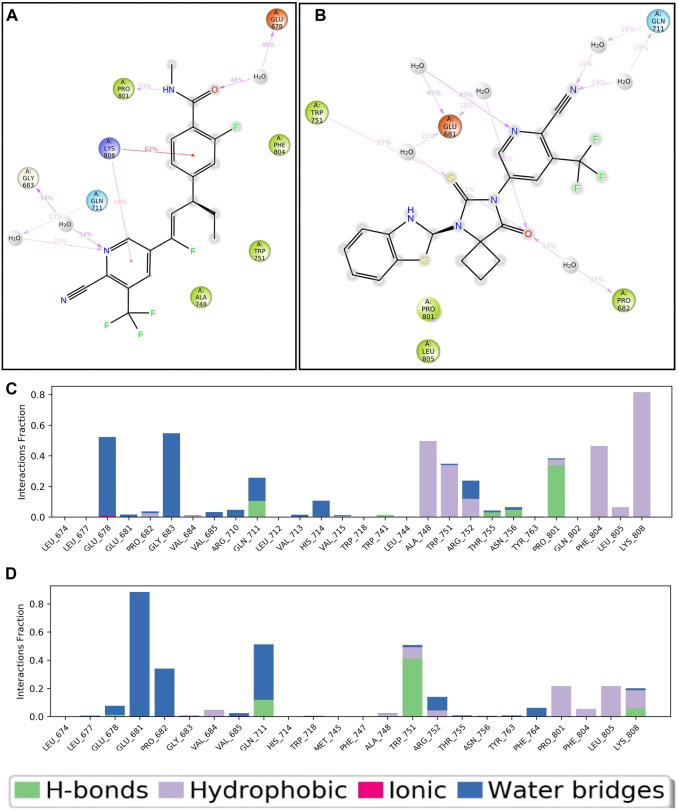
Two-dimensional interaction of APL43 **(A)** and APL80 **(B)** with respect to the protein during 100–nS MD simulation; Histogram represents the hydrogen bonding interactions of APL43 **(C)** and APL80 **(D)** with respect to residues of protein.

Throughout the entire simulation process, it is crucial to understand the atoms' interactions with each other in order to predict how the protein and ligand will bind. Throughout the entire 100 nS simulation, we examined numerous binding interactions between the protein and ligand molecules. These included hydrogen bonds, ionic interactions, hydrophobic contact, and the salt bridge. This study demonstrates the involvement of numerous intramolecular interactions, including hydrogen bonds, phosphorylation, and water molecules in water bridges. Figure 5A depicts the ligand-APL43 interaction with protein amino acids and other relevant fragments. Even though we have not noticed any direct interaction with carbon molecules, the interaction with the N, O, and NH groups formed hydrophilic, hydrophobic, and hydrogen bonding interactions with respective percentiles. Furthermore, the direction of the arrows shows both donors and acceptors. The H_2_O molecules interacted widely, forming water bridges, while the amino acids interacted directly, as well as through hydrophilic and other interactions. There are three water molecules involved in the interaction, along with GLU678, GLY683, and GLN711. The GLN711 showed a hydrogen bond, LYS808 forms a pi-cation bond with the phenyl and pyridinyl rings, PRO801 forms a single hydrophobic bond, and GLU678 forms a pi-anion bond (Figure 5A).

**FIGURE 6:**
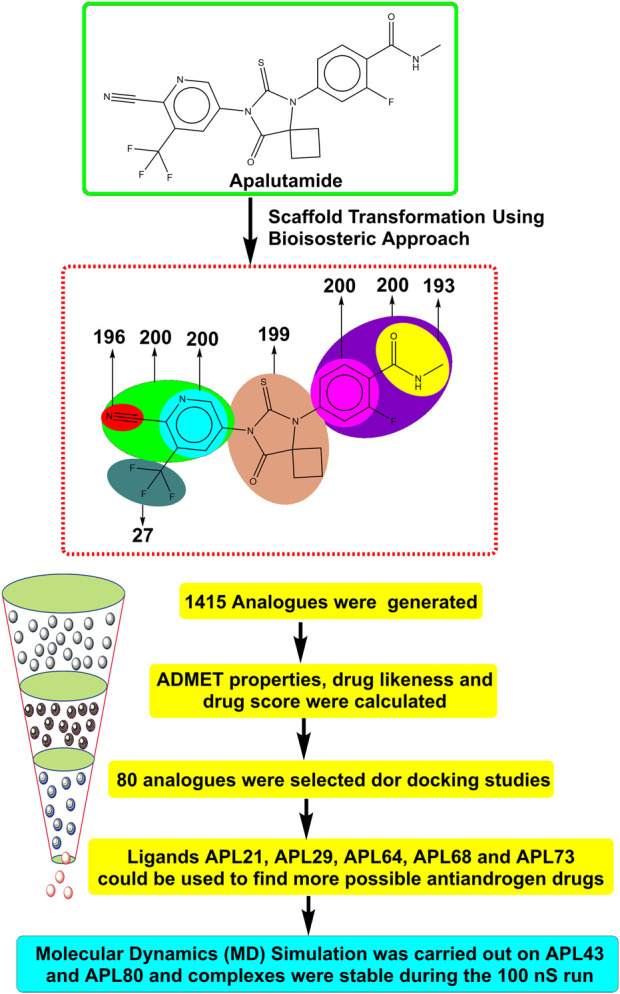
Overall workflow of the present study.

Figure 5B shows the APL80 ligand interaction with protein amino acids. The interaction with the O, S, and N groups resulted in hydrophilic, hydrophobic, and hydrogen bonding interactions with respective percentiles. Furthermore, the direction of the arrows shows both donors and acceptors. The H_2_O molecules interacted widely, forming water bridges, while the amino acids interacted directly, as well as through hydrophilic and other interactions. There are six water molecules involved in the interaction, along with GLU681, TRP751, PRO682, and GLN711. GLN711 formed the hydrogen bond, GLU681 made the pi-anion bond, and PRO682 and TRP751 made the hydrophobic bond. During 100 nS run, we displayed the percentiles of interaction for each type of bond. Figures 5C, D show more statistical explanations by dividing protein-ligand interactions (contacts) into four groups: ionic, hydrophobic, hydrogen bonds, and water bridges. Overall, the MD simulation results suggested that both complexes are stable and have lower RMDS, RMSF, and good interactions. The overall workflow of the study is summarized in Figure 6.

## 4 Conclusion

APL is one of the non-steroidal antiandrogen drug used in the management of PC. During therapy with APL, it causes several toxicities. So, structure modification of APL is required to get novel and less toxic analogues. By using the bioisosteric approach, *in silico* design of APL analogues was carried out using MolOpt. We selected newer bioisosteres of APL to calculate the pharmacokinetic and toxicological properties using ADMETlab 2.0. Additionally, the DL and DS scores of the designed analogues of APL were also computed using PEO. Docking studies of selected analogues were also carried out using ADV and Discovery Studio. Analogues APL19, APL35, APL43, APL76, and APL80 have shown good interactions with the protein (PDB ID: *5T8E*), which is similar to the standard drug (APL). The common amino acid residues ARG752, GLN711, THR755, GLU681, and PRO682 might play a crucial role in the binding affinity and antagonistic activity of androgen receptors. The molecular docking study has shown promising results with many ligands, such as APL19, APL35, APL43, APL76, and APL80. We ran the MD simulation on two ligands. We only selected the top two ligands for further simulation using the SPC water model. The MD simulation results for both ligands, APL43 and APL80, were promising. According to the data obtained from ADMET, DL, DS score, and docking studies, the ligands APL19, APL35, APL43, APL76, and APL80 can be used as potential antiandrogen agents for the treatment of prostate cancer. Further work is in progress in order to evaluate this hypothesis as an antiandrogen agent in the management of PC.

## Data Availability

The original contributions presented in the study are included in the article/Supplementary Material, further inquiries can be directed to the corresponding author.
