# Supplementary material for: Exploring novel Apalutamide analogues as potential therapeutics for prostate cancer: design, molecular docking investigations and molecular dynamics simulation
Source: Front Chem. 2024 Aug 6;12:1418975. doi: 10.3389/fchem.2024.1418975 (PMC11333239; doi:10.3389/fchem.2024.1418975)
Supplement: Supplementary file 1 [file Table1.docx]

**Table S1. Structures of the selected apalutamide analogues and their physico-chemical properties.**

| **S. No.** | **Entry No.** | **Structure** | **MW** | **nHA** | **nHD** | **LogP** | **TPSA** |
| --- | --- | --- | --- | --- | --- | --- | --- |
| 1 | APL1 |  | 464.06 | 7 | 1 | 3.084 | 97.53 |
| 2 | APL2 |  | 480.09 | 7 | 2 | 2.173 | 100.69 |
| 3 | APL3 |  | 480.09 | 7 | 2 | 2.173 | 100.69 |
| 4 | APL4 |  | 488.1 | 7 | 1 | 2.857 | 84.62 |
| 5 | APL5 |  | 479.05 | 6 | 2 | 2.861 | 86.25 |
| 6 | APL6 |  | 477.12 | 6 | 1 | 3.127 | 72.26 |
| 7 | APL7 |  | 478.08 | 8 | 3 | 2.443 | 115.35 |
| 8 | APL8 |  | 435.08 | 6 | 2 | 2.763 | 86.25 |
| 9 | APL9 |  | 483.14 | 7 | 1 | 2.083 | 89.33 |
| 10 | APL10 |  | 494.08 | 9 | 1 | 0.835 | 111.33 |
| 11 | APL11 |  | 481.09 | 9 | 1 | 1.362 | 107.15 |
| 12 | APL12 |  | 479.08 | 9 | 1 | 1.658 | 115.11 |
| 13 | APL13 |  | 480.1 | 8 | 1 | 2.246 | 94.26 |
| 14 | APL14 |  | 467.08 | 9 | 1 | 1.616 | 107.15 |
| 15 | APL15 |  | 467.14 | 9 | 1 | 1.18 | 95.81 |
| 16 | APL16 |  | 492.16 | 8 | 1 | 1.937 | 92.57 |
| 17 | APL17 |  | 491.16 | 7 | 1 | 2.661 | 89.33 |
| 18 | APL18 |  | 477.14 | 7 | 1 | 2.326 | 89.33 |
| 19 | APL19 |  | 449.11 | 7 | 1 | 1.81 | 89.33 |
| 20 | APL20 |  | 463.13 | 7 | 1 | 2.034 | 89.33 |
| 21 | APL21 |  | 466.14 | 8 | 1 | 1.325 | 92.57 |
| 22 | APL22 |  | 466.14 | 8 | 1 | 1.871 | 92.57 |
| 23 | APL23 |  | 466.14 | 8 | 2 | 1.317 | 101.36 |
| 24 | APL24 |  | 468.12 | 9 | 2 | 1.004 | 110.59 |
| 25 | APL25 |  | 468.12 | 9 | 2 | 0.837 | 110.59 |
| 26 | APL26 |  | 500.12 | 8 | 2 | 2.252 | 101.36 |
| 27 | APL27 |  | 479.1 | 7 | 1 | 2.642 | 89.33 |
| 28 | APL28 |  | 477.09 | 7 | 1 | 2.458 | 89.33 |
| 29 | APL29 |  | 491.1 | 7 | 1 | 2.869 | 89.33 |
| 30 | APL30 |  | 463.07 | 7 | 1 | 2.15 | 89.33 |
| 31 | APL31 |  | 465.09 | 7 | 1 | 2.28 | 89.33 |
| 32 | APL32 |  | 462.17 | 6 | 1 | 3.294 | 86.09 |
| 33 | APL33 |  | 507.14 | 7 | 1 | 3.424 | 89.33 |
| 34 | APL34 |  | 449.15 | 7 | 2 | 2.004 | 98.12 |
| 35 | APL35 |  | 434.14 | 6 | 1 | 2.613 | 86.09 |
| 36 | APL36 |  | 448.15 | 6 | 1 | 3.05 | 86.09 |
| 37 | APL37 |  | 437.06 | 7 | 2 | 1.937 | 95.87 |
| 38 | APL38 |  | 448.15 | 6 | 1 | 3.043 | 86.09 |
| 39 | APL39 |  | 493.12 | 7 | 1 | 2.732 | 89.33 |
| 40 | APL40 |  | 449.11 | 8 | 1 | 2.301 | 106.4 |
| 41 | APL41 |  | 435.1 | 8 | 2 | 1.899 | 112.94 |
| 42 | APL42 |  | 449.11 | 8 | 1 | 2.277 | 106.4 |
| 43 | APL43 |  | 409.12 | 4 | 1 | 4.105 | 65.78 |
| 44 | APL44 |  | 421.08 | 8 | 2 | 1.788 | 112.94 |
| 45 | APL45 |  | 442.07 | 7 | 1 | 1.975 | 93.51 |
| 46 | APL46 | `  | 480.1 | 8 | 1 | 2.04 | 94.26 |
| 47 | APL47 |  | 482.08 | 9 | 1 | 2.701 | 103.37 |
| 48 | APL48 |  | 493.08 | 8 | 1 | 2.431 | 98.44 |
| 49 | APL49 |  | 493.08 | 8 | 1 | 2.281 | 98.56 |
| 50 | APL50 |  | 481.12 | 7 | 2 | 2.955 | 80.8 |
| 51 | APL51 |  | 482.11 | 8 | 2 | 2.585 | 93.69 |
| 52 | APL52 |  | 466.11 | 6 | 1 | 2.726 | 65.54 |
| 53 | APL53 |  | 466.11 | 6 | 1 | 2.716 | 65.54 |
| 54 | APL54 |  | 468.09 | 7 | 2 | 1.968 | 85.51 |
| 55 | APL55 |  | 452.09 | 6 | 1 | 2.453 | 65.54 |
| 56 | APL56 |  | 452.09 | 6 | 1 | 2.441 | 65.54 |
| 57 | APL57 |  | 480.12 | 6 | 1 | 2.794 | 65.54 |
| 58 | APL58 |  | 468.09 | 7 | 2 | 2.504 | 85.77 |
| 59 | APL59 |  | 453.09 | 7 | 1 | 2.428 | 78.43 |
| 60 | APL60 |  | 453.09 | 7 | 1 | 2.289 | 78.43 |
| 61 | APL61 |  | 468.09 | 7 | 1 | 2.449 | 74.77 |
| 62 | APL62 |  | 483.1 | 8 | 1 | 2.316 | 87.66 |
| 63 | APL63 |  | 491.1 | 7 | 1 | 2.421 | 89.33 |
| 64 | APL64 |  | 496.12 | 7 | 1 | 2.277 | 74.77 |
| 65 | APL65 |  | 495.14 | 7 | 2 | 3.161 | 80.8 |
| 66 | APL66 |  | 496.12 | 7 | 2 | 2.244 | 85.77 |
| 67 | APL67 |  | 492.12 | 6 | 1 | 3.598 | 65.54 |
| 68 | APL68 |  | 491.1 | 7 | 1 | 2.421 | 89.33 |
| 69 | APL69 |  | 494.1 | 7 | 1 | 2.288 | 82.61 |
| 70 | APL70 |  | 558.1 | 8 | 1 | 2.649 | 99.68 |
| 71 | APL71 |  | 497.08 | 9 | 1 | 2.5 | 108.68 |
| 72 | APL72 |  | 536.08 | 7 | 1 | 4.151 | 74.77 |
| 73 | APL73 |  | 482.1 | 7 | 1 | 3.054 | 74.77 |
| 74 | APL74 |  | 534.05 | 8 | 1 | 2.616 | 99.68 |
| 75 | APL75 |  | 441.11 | 7 | 1 | 1.76 | 89.33 |
| 76 | APL76 |  | 459.1 | 7 | 1 | 2.029 | 89.33 |
| 77 | APL77 |  | 412.08 | 7 | 0 | 2.22 | 86.53 |
| 78 | APL78 |  | 396.09 | 6 | 0 | 2.42 | 77.3 |
| 79 | APL79 |  | 428.09 | 5 | 0 | 3.39 | 60.23 |
| 80 | APL80 |  | 461.06 | 6 | 1 | 3.681 | 72.26 |
| STD | APL |  | 477.09 | 7 | 1 | 2.458 | 89.33 |

MW, molecular weight; nHA, number of hydrogen bond acceptor; nHD, number of hydrogen bond donor; nRot, number of rotatable bonds; TPSA, topological polar surface area; logP, the logarithm of partition coefficient value; STD, Standard; APL, Apalutamide.
